# Supplementary material for: A senataxin-associated exonuclease SAN1 is required for resistance to DNA interstrand cross-links
Source: Nat Commun. 2018 Jul 3;9:2592. doi: 10.1038/s41467-018-05008-8 (PMC6030175; doi:10.1038/s41467-018-05008-8)
Supplement: Supplementary file 2 — Supplementary Information [file 41467_2018_5008_MOESM2_ESM.pdf]

A Senataxin-Associated Exonuclease, SAN1, Is Required For Resistance To DNA Interstrand Cross-links

Andrews et al.

Supplementary Figure 1 Andrews et al.

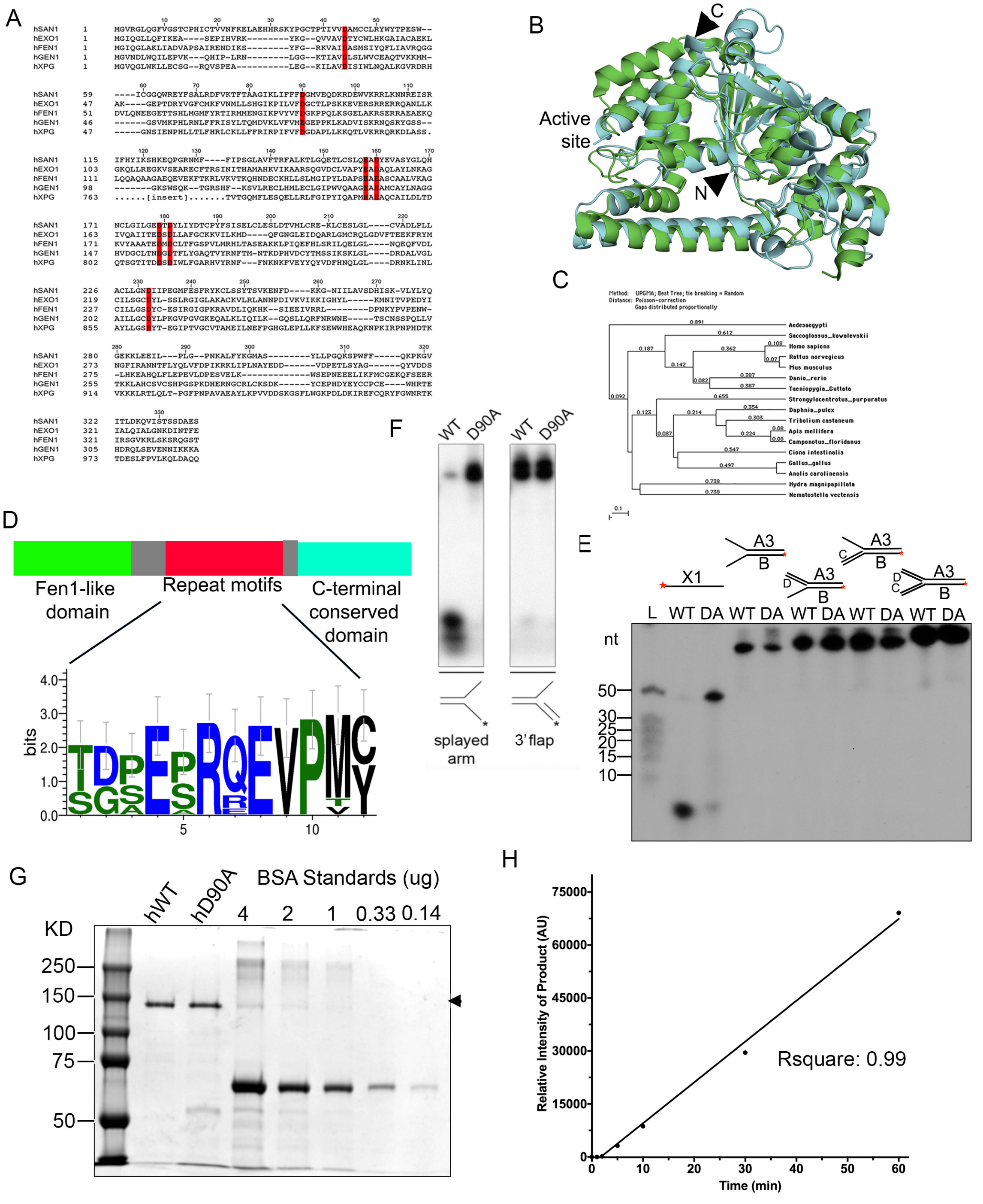

Supplementary Figure 1. (a) Sequence alignment of nuclease domains of human SAN1, EXO1, FEN1, GEN1 and XPG. Identical and similar residues are in bold. Conserved acidic residues in the active site are highlighted in red**.** (b) The nuclease domain sequence of SAN1 was submitted to the Robetta server (http://robetta.bakerlab.org) and the server found a confident match to the A. fulgidus FEN1 protein, whose structure was used as a template for comparative modeling. SAN1 model (light blue) and FEN1 template (PDB

1RXW) (green) were aligned and visualized with the PyMol Molecular Graphics System, Version 1.3 Schrödinger, LLC. (c) Phylogenetic analysis of SAN1 gene in Metazoa. MacVector was used to generate the tree. (d) Schematic of the domain architecture of SAN1 including the N-terminal nuclease domain, central repeat region of around twelve 15-amino acid repeats, and conserved C-terminus. (e) SAN1 WT or SAN1 DA incubated with splayed duplex, 3' flap, 5' flap, and replication fork structures where the bottom strand (B) is 5' ^{32}P labeled. (f) SAN1 WT or SAN1 DA incubated with a 5' ^{32}P splayed arm and 3' flap structure. (g) Affinity purified hWT SAN1 and hDA SAN1 from 293T cells as compared to BSA standards visualized by Coomassie brilliant blue stain. Arrow shows hSAN1 expected size of 150 kD. (h) Time course of SAN1 WT in linear range of the nuclease reaction with ssDNA X1 as a substrate.

Supplementary Figure 2 Andrews et al.

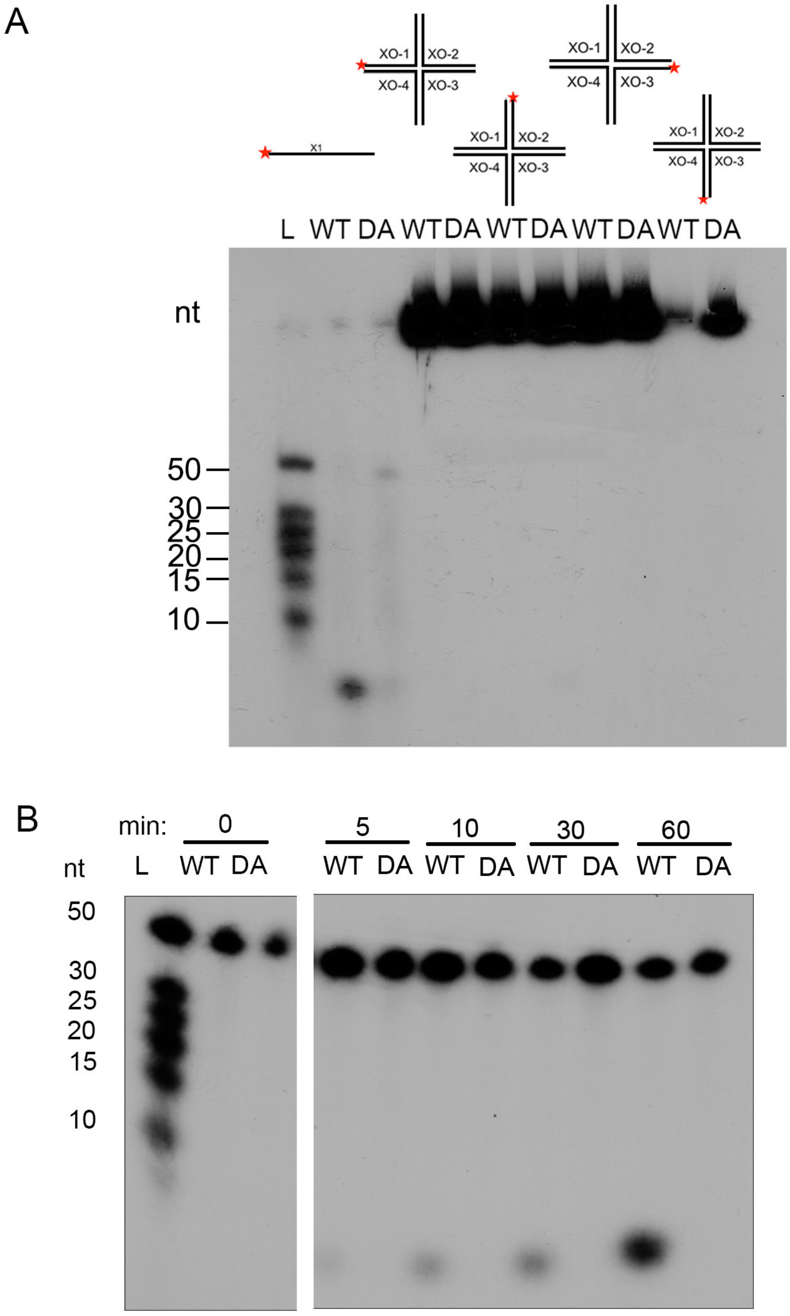

Supplementary Figure 2. (a) Affinity purified FLAG-tagged WT or DA SAN1 from 293T cells was incubated 5’ ^{32}P labeled Holliday Junction structures and products were separated by denaturing gel electrophoresis and ^{32}P fragments were detected by autoradiography. (b) Time course of 5’ ^{32}P-labeled ssDNA X1 digest.

Supplementary Figure 3 Andrews et al.

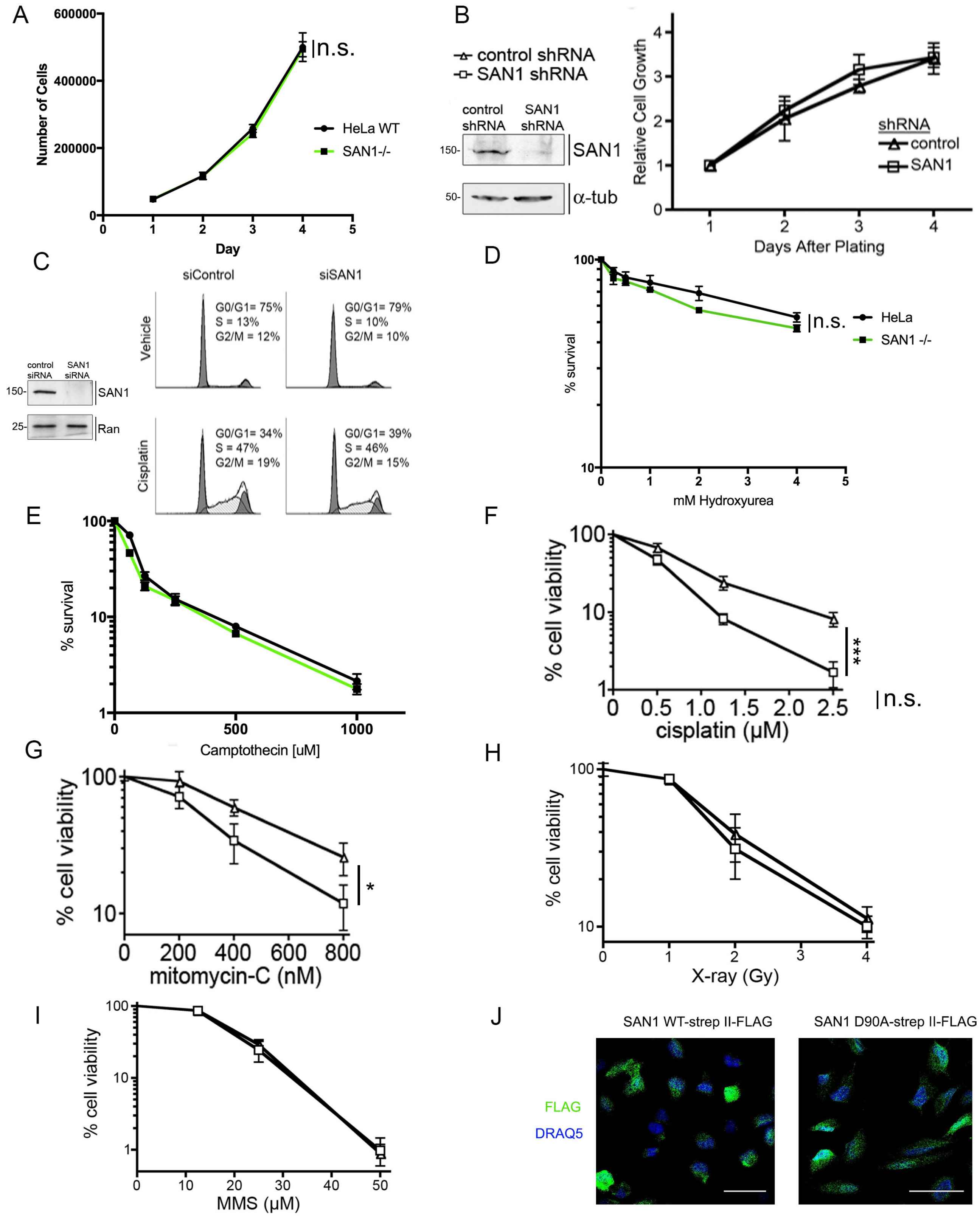

Supplementary Figure 3. (a) Proliferation assay of WT HeLa cells and SAN1$^{-/-}$ HeLa cells generated by

CRISPR/Cas9 technology. Cells were plated in triplicate on day 0 and were trypsinized and counted each day for 4 days. Error bars represent SEM. (b) Knockdown of SAN1 has no significant effect on cell proliferation. Immunoblot for control vs. shRNA treated HeLa cells (left panel). α-tubulin was used as a loading control. (c) Cell cycle distribution of cells treated with SAN1 or control siRNA and vehicle or Cisplatin. Immunoblot (left panel) shows knockdown of SAN1 by siRNA. Ran was used as a loading control. Clonogenic survival assays of WT HeLa cells compared to SAN1-/- HeLa cells after treatment with (d) hydroxyurea and (e) camptothecin. Statistical significance for CSAs was determined by two-way ANOVA test. Error bars denote s.e.m. * = $p<0.05$, ** = $p<0.01$, *** = $p<0.001$, **** = $p<0.0001$. (f-i) HeLa cells were transfected with shRNA against SAN1 or a scrambled sequence as a control, and colony survival assays were performed in response to Cisplatin, MMC, ionizing radiation, or methyl methanesulfonate (MMS). Statistical significance was determined by unpaired two-tailed t-test. Efficiency of silencing is shown in (b). (j) Immunofluorescence staining of SAN1 WT and D90A –Strep$_2$-FLAG tagged cells showing similar cytoplasmic and nuclear localizations. Scale bar = 50 μm.

Supplementary Figure 4 Andrews et al.

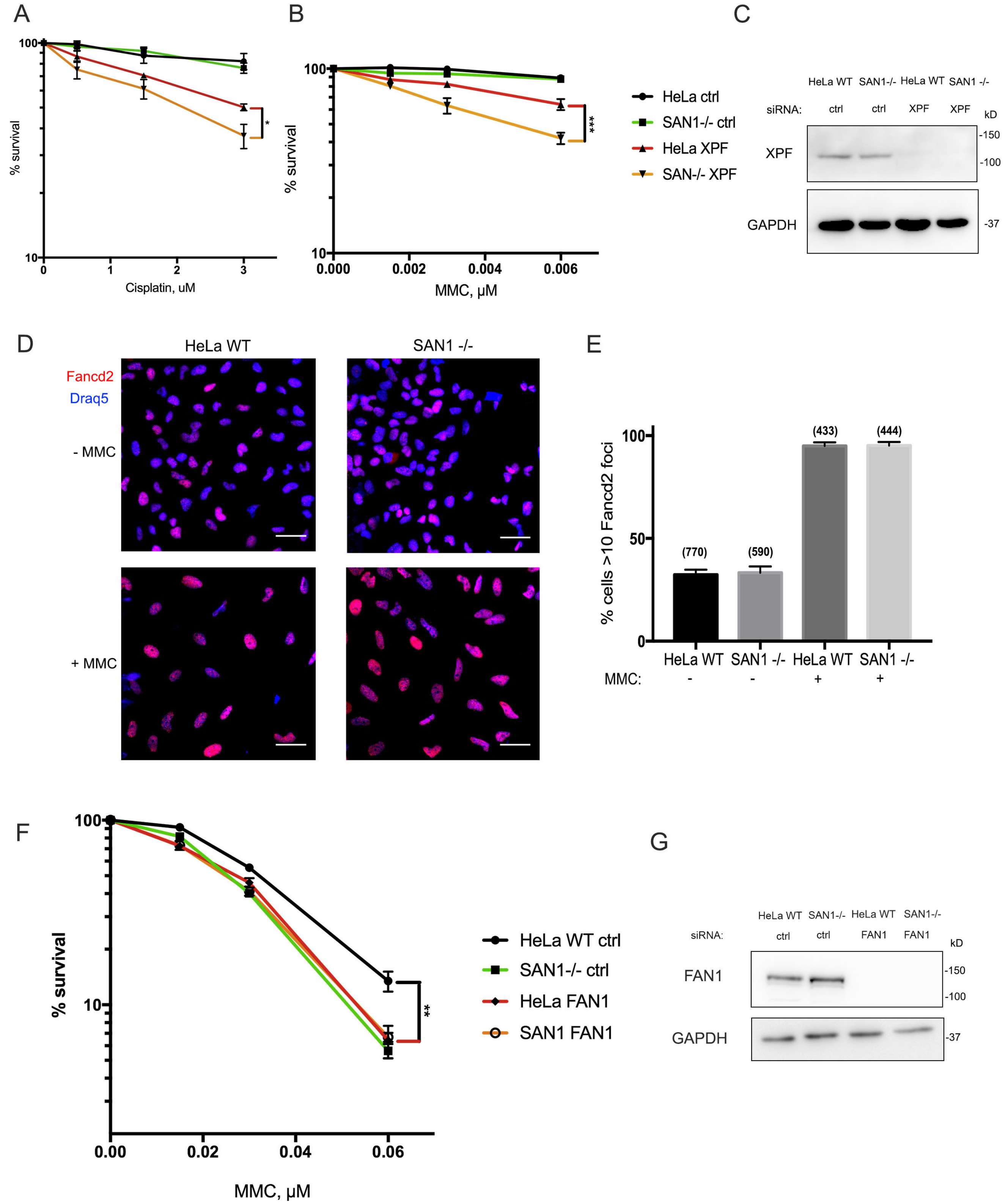

Supplementary Figure 4. (a,b) CSAs of HeLa WT and SAN1 -/- cells treated with scrambled ctrl siRNA or XPF

siRNA, in response to Cisplatin and MMC (N=3) Statistical significance shown between HeLa WT and SAN1-/- +XPF siRNA conditions determined by two-way ANOVA. Error bars denote s.e.m. * = $p<0.05$, ** = $p<0.01$, *** = $p<0.001$, **** = $p<0.0001$. (c) Immunoblot showing siRNA knockdown of XPF in HeLa WT and SAN1-/- cells. (d) Immunofluorescence images displaying FANCD2 foci formation in HeLa WT and SAN1-/- cells treated with vehicle or MMC. Scale bar = 50 μm. (e) Quantification of the percentage of cells with >10 FANCD2 foci in HeLa WT and SAN1-/- cells treated with vehicle or 0.045 μM MMC. (N=3 biological replicates, at least 100 cells were analyzed per sample. (f) CSA of HeLa WT and SAN1-/- cells treated with scrambled ctrl siRNA or FAN1 siRNA, in response to MMC (N=3) Statistical significance shown between HeLa WT +ctrl siRNA and HeLa WT +FAN1 siRNA conditions determined by two-way ANOVA. (g) Immunoblot showing siRNA knockdown of FAN1 in HeLa WT and SAN1-/- cells.

Supplementary Figure 5 Andrews et al.

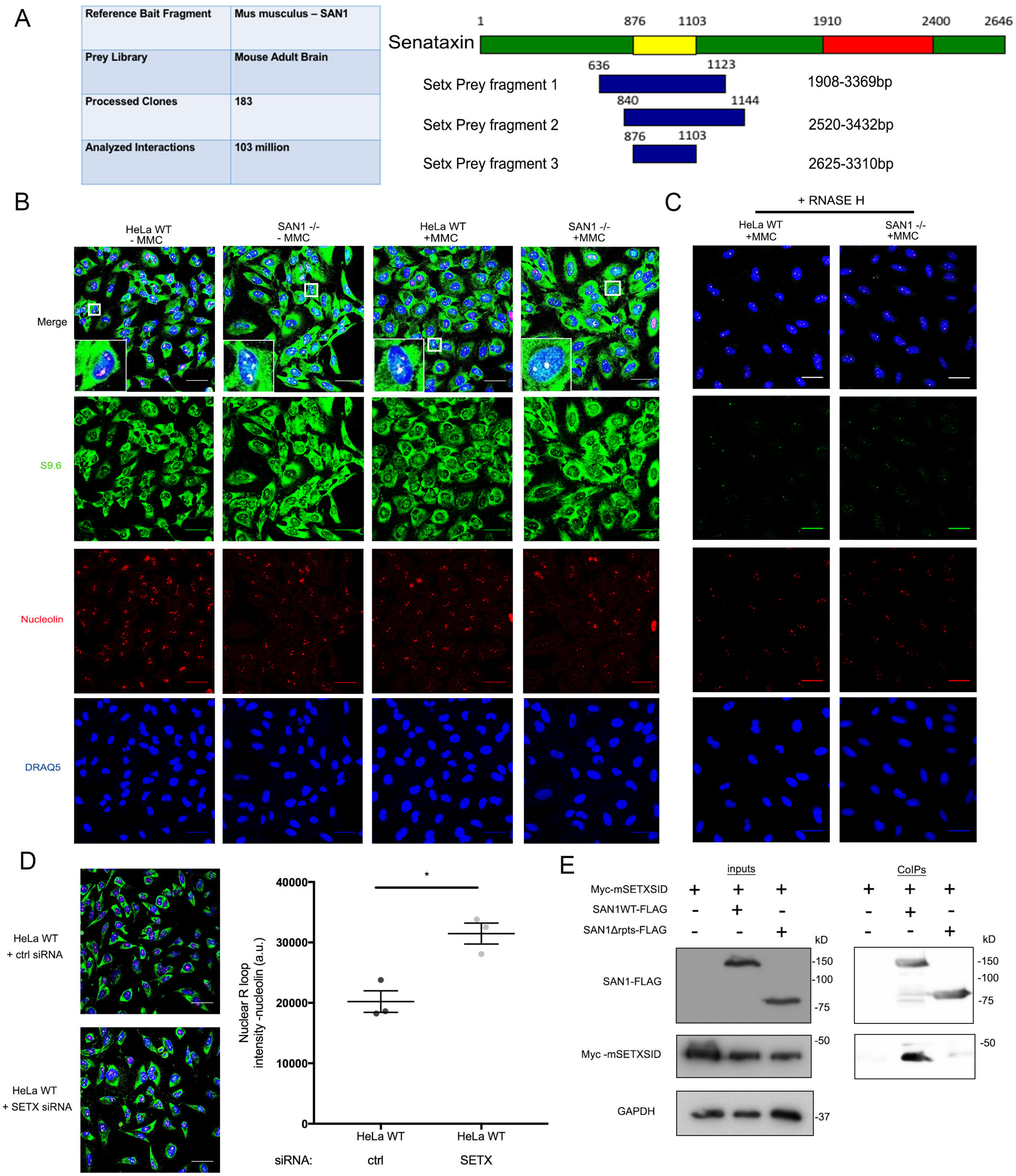

Supplementary Figure 5. (A) Yeast two-hybrid (Y2H) screen and library information in addition to a schematic

detailing mSETX prey fragments that interacted with mSAN1 in Y2H screen. The shared interaction domain of mSETX prey fragments shows that SAN1 interacts with SETX in a region of the N-terminus and not near the RNA/DNA helicase domain at the C-terminus. (B) HeLa WT and SAN1-/- cells were treated with vehicle or 1 μM MMC and labeled with a monoclonal antibody to detect R-loops (S9.6), a polyclonal antibody against nucleolin, and Draq5 to label DNA. Intensity of nuclear R-loop staining was quantified from the nucleus following masking with the DRAQ5 channel, and subtraction from nucleolin regions. (C) Validation of S9.6 R-loop antibody specificity by pre-treatment with R-loop degrading enzyme RNAse H in HeLa WT and SAN1-/- cells treated with 1 μM MMC. (D) Left panel: Immunofluorescence images of HeLa WT cells transfected with ctrl or SETX siRNA and stained with a monoclonal antibody to detect R-loops (S9.6), a polyclonal antibody against nucleolin, and Draq5 to label DNA. Right panel: Quantification of R-loop intensity as in Figure 7I. Statistical significance determined by t-test with Welch's correction (N=3 biological replicates, at least 70 cells were analyzed per sample). (E) Mapping of the binding region of hSAN1 to the shared interaction domain (SID) of mSETX determined by the Y2H library fragments. Cells were transfected with myc-mSETXSID and either empty vector, SAN1WT-FLAG, or SAN1ΔRep-FLAG. SAN1WT-FLAG or SAN1ΔRep-FLAG were co-immunoprecipitated and lysates were blotted for FLAG and Myc.

Supplementary Figure 6 Andrews et al.

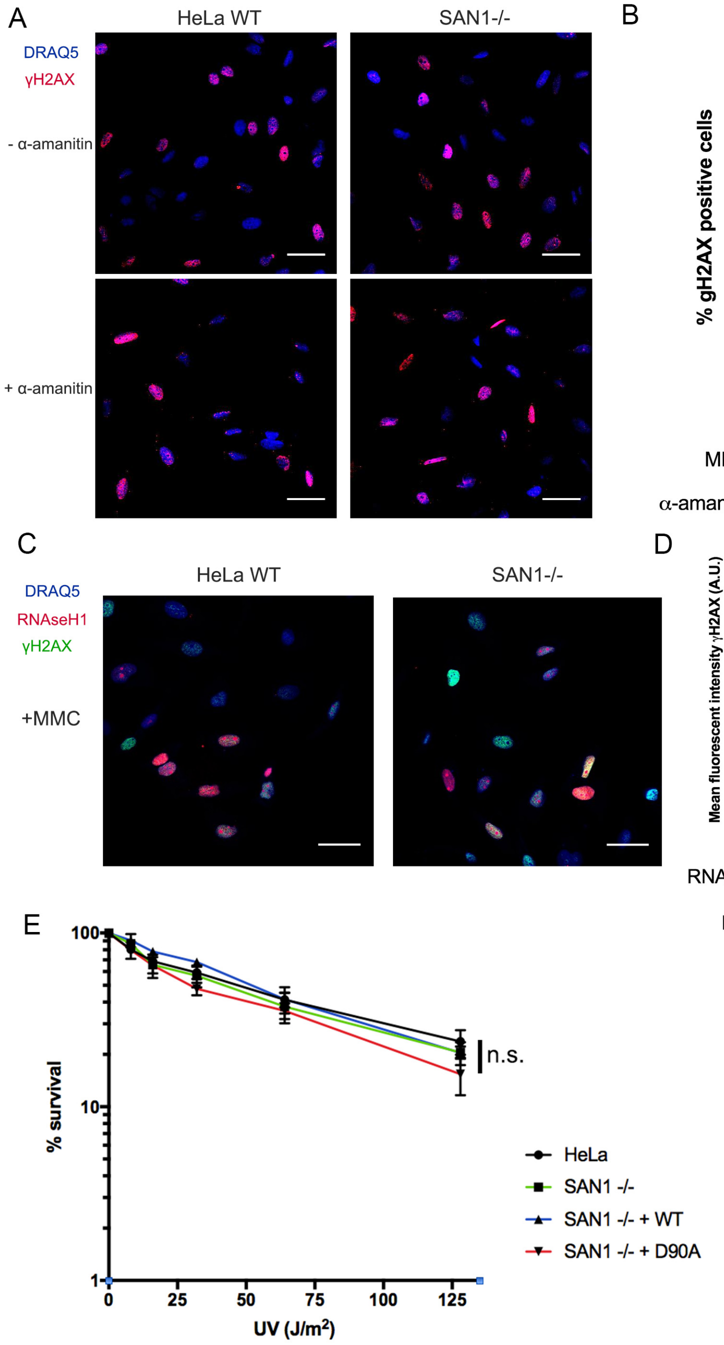

Supplementary Figure 6. (a) Immunofluorescence images of cells treated with 10 μM MMC and vehicle or 50μg/mL of α-amanitin for 6 hrs to selectively inhibit RNA pol II, then stained for γH2AX and DNA (DRAQ). (b) Quantification of data in (a) measuring the percentage of cells positive for γH2AX staining (N=3 biological replicates, at least 200 cells per sample were analyzed). (c) Representative immunofluorescence image of cells expressing RNaseH1-NLS-mCherry and treated with 1μM MMC for 30 hrs, then stained for γH2AX and DNA. (d)

Quantification of data in (C) measuring the mean fluorescent intensity of γH2AX in cells positive and negative for RNaseH1-NLS-mCherry (N=3 biological replicates, at least 60 cells per sample were analyzed). Statistical significance determined by t-test with Welch's correction. (e) Colony survival assay of HeLa WT, SAN1-/-, SAN1-/- +WT and SAN1-/- +D90A cell lines following exposure to UV.

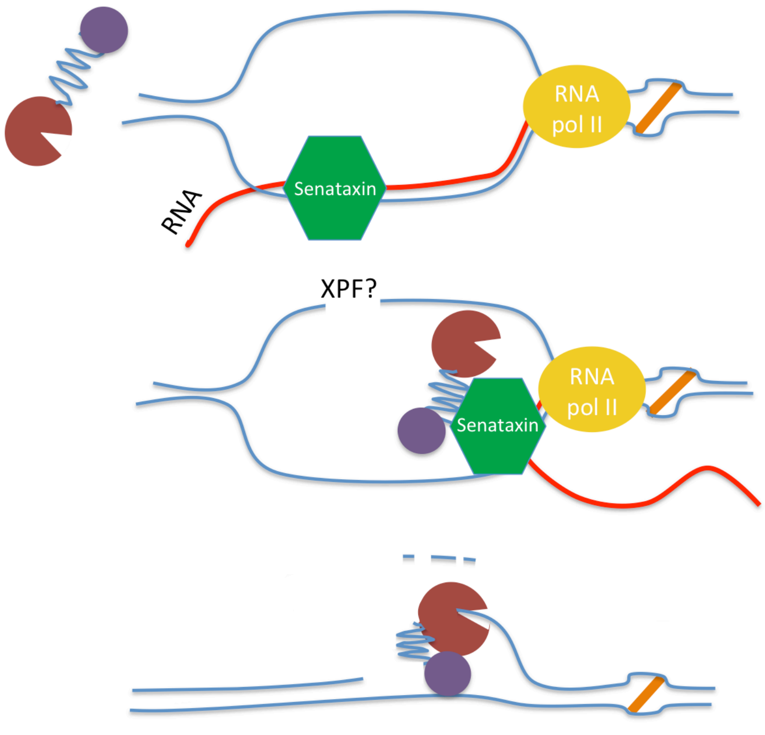

Supplementary Figure 7. Speculative model for SAN1 function with SETX in processing of ICLs. Collision of transcription complexes with an ICL results in the formation of R-loops. The helicase SETX is recruited to the RNA/DNA hybrid and unwinds it. SETX also recruits the SAN1 nuclease. If an incision is generated in the ssDNA loop, perhaps by XPF, SAN1 can digest the free 5' end back to the ICL, where other nucleases participate in unhooking of the lesion.

Supplementary Figure 8 Andrews et al.

Figure 3B

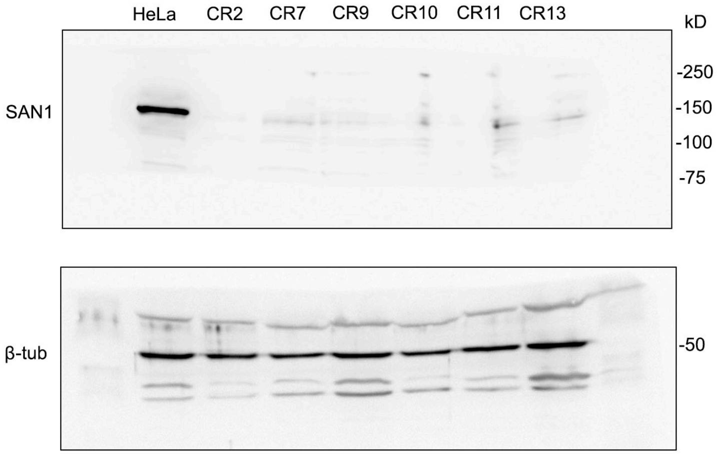

Figure 3F

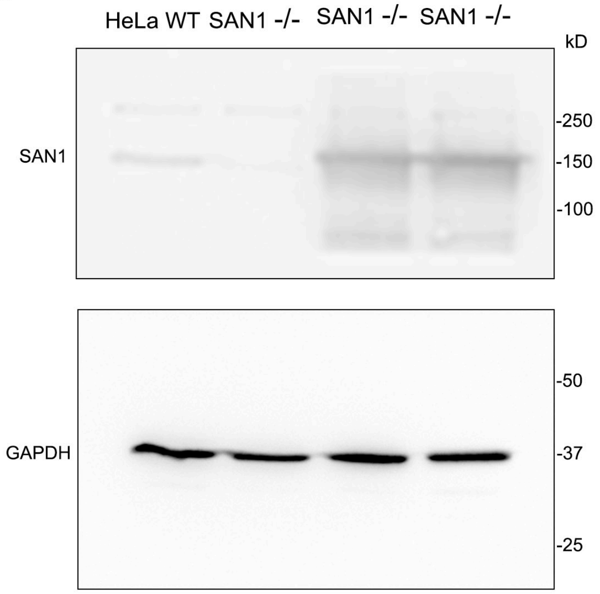

Figure 3J

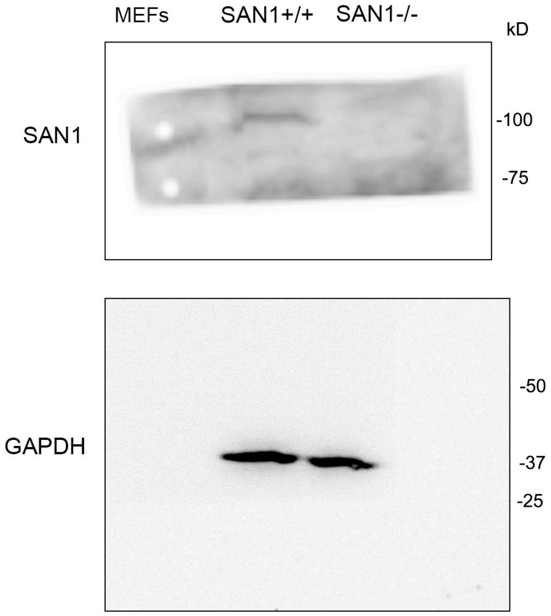

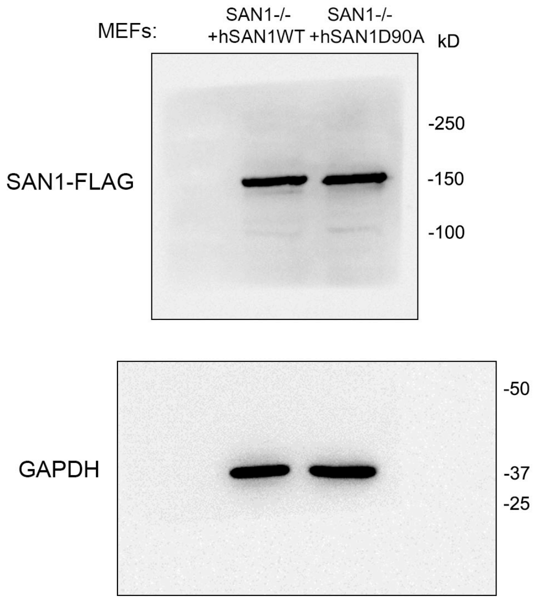

Supplementary Figure 8. Raw western blot data from Figure 3. Top left panels: SAN1-/- HeLa cell clones

generated using CRISPR/CAS9 and loading controls as shown in Figure 3B. Top right panels SAN1WT and SAN1D90A rescue lines and loading control as shown in Figure 3F. Bottom left panels: SAN1+/+ and SAN1-/- MEFs raw western blots from Figure 3J. Bottom right panels: MEF rescue cell line raw western blots and loading control from Figure 3J.
